# Prenatal Psychological Stress Exposure and Neurodevelopment and Health of Children

**DOI:** 10.3390/ijerph16193657

**Published:** 2019-09-29

**Authors:** Laura S. Bleker, Susanne R. de Rooij, Tessa J. Roseboom

**Affiliations:** Department of Obstetrics and Gynaecology, Department of Clinical Epidemiology, Biostatistics and Bioinformatics, Amsterdam University Medical Centers, Meibergdreef 9, 1105 AZ Amsterdam, The Netherlands; s.r.derooij@amsterdamumc.nl (S.R.d.R.); t.j.roseboom@amsterdamumc.nl (T.J.R.)

## 1. Introduction

Depression and anxiety are highly prevalent in pregnancy, with an estimated prevalence of 12% for depression [1], and 15.2% for anxiety [2]. Also, mild to moderate stress is reported by a third to more than half of respondents in healthy populations of pregnant women across the globe [3,4,5,6,7,8]. Perinatal mental health problems impact maternal wellbeing and that of her direct social environment to a great extent. Moreover, early-life exposure of children to maternal psychopathology or stress, either chronic or acutely induced by a traumatic experience during in utero development or in the first years of life, could have long-lasting consequences [9,10]. Prenatal exposure to an adverse environment may have an effect on fetal development through physiological intrauterine mechanisms, while the postnatal environment may adversely affect child development through the influence of negative parental behaviors and (lack of) care [11]. Besides a potential direct biological effect of maternal stress on fetal and/or child neurodevelopment, many environmental and socioeconomic factors may interact with prenatal depression, anxiety and/or stress in affecting child neurodevelopment. The environment can act as a buffer for maternal stress or it can increase maternal stress and its effects on child neurodevelopment, accordingly. This is illustrated by the observation that chronic stress, depression, and anxiety in the prenatal period are consistently more prevalent in low- and middle-income countries (LMIC) [12,13]. Poverty, war, and domestic and/or sexual violence that occur more often in LMIC all contribute to the increased levels of stress, depression, and anxiety described in pregnant women [14,15]. Knowledge about the actual moderating role of such risk or protective factors is important to be able to identify women at highest risk for stress or psychopathology during the perinatal period, and to tailor interventions in such a way that they benefit both mother and child.

## 2. Objective of the Special Issue

We are pleased to introduce a Special Issue of the International Journal of Environmental Research and Public Health on prenatal psychological stress exposure and subsequent neurodevelopment and health of children. Research on the effects of prenatal stress, depression, and anxiety on child neurodevelopment is complicated by the numerous factors that influence both maternal mood and child development. It is therefore particularly challenging to identify the independent contribution of maternal mood to the child’s neurodevelopment. The collection of international cohort studies and (pilot) randomized controlled trials (RCTs) presented in this Special Issue contribute to a better understanding of the various socio-economic-environmental factors that play a role in early life stress, depression, and anxiety, and through which mechanisms these symptoms may affect child neurodevelopment.

## 3. The Papers

Mclean et al. investigated the effects of various aspects of stress in pregnancy, as brought about by a severe flood, on maternal reports of 16-month toddler temperament, four-year maternal-reported childhood anxiety symptomatology, and teacher reports of internalizing behaviors [16]. Severity of maternal hardship during pregnancy was uniquely associated with anxiety symptoms of the child at four years of age. Mediation analyses showed that higher levels of child negative reactivity at 16 months accounted, in part, for the relationship between increased maternal flood-related hardship and greater internalizing behaviors (maternal but not teacher report). Neither child sex nor gestational timing of exposure moderated the mediations. These findings highlight the several pathways through which various aspects of (disaster-related) prenatal maternal stress may influence early childhood anxiety symptomatology. Faleschini et al. examined the association between prenatal and postnatal maternal mood and child development [17]. Strong features of this study are the inclusion of maternal IQ as covariate, and a dual assessment of child development by both a parent and a teacher, thereby limiting the potential confounding effect of parental mood on the interpretation of the child’s development. High prenatal maternal depressive symptoms predicted poorer behavioral executive functions and higher levels of behavioral difficulties in children using teachers’ and mothers’ reports, highlighting the crucial role of prenatal depression in later child development. A factor that has an impact on pre- and postnatal mental health and child development is sleep quality during pregnancy. Studies have shown that poor sleep quality during pregnancy is associated with higher antepartum and postpartum depression and adverse birth outcomes [18,19,20]. Bais et al. explored both subjective and objective sleep parameters and their effects on the course of antepartum depressive symptoms in a sample of 18 psychiatric patients [21]. They found that objectively measured sleep (by use of a wrist-worn recorder that measured the number of movements during sleep), total Pittsburgh Sleep Quality Index (PSQI) score (both continuous and dichotomous), and four PSQI subscales (sleep quality, sleep duration, sleep disturbances, and daytime dysfunctions) were all moderately to strongly correlated with depressive symptoms during pregnancy. Some sleep parameters were associated with the course of antepartum depressive symptoms during the third trimester, both unadjusted and adjusted for Edinburgh Postnatal Depression Score (EPDS) score at the start, and other potential confounders (medication, treatment, psychiatric diagnosis, age, education level, parity, work, and ethnicity). This suggests that women who report more depressive symptoms also suffer from specific sleep problems. The authors conclude that further research is necessary to explore the causal link between sleep problems and antepartum depressive symptoms found in psychiatric patients. The role of social support in the perinatal period has also been of particular interest as a possible protective factor for coping difficulties arising from the many challenges that motherhood brings [22,23,24,25]. Milgrom and colleagues investigated the trajectory of the relationships between perceived social support during pregnancy on depression and anxiety symptoms during pregnancy and postpartum [26]. They additionally examined the influence of social support on child development and parenting-related stress. Results suggested that during the period towards childbirth, the impact of prior and current social support on pregnant women’s psychological wellbeing becomes increasingly important, although there was no evidence that the relationship between depression or anxiety and child outcomes was mediated by social support. In a similar fashion, Aris-Meijer and colleagues investigated to what extent specific life events during pregnancy, delivery complications, unfavorable obstetric outcomes, and antenatal levels of anxiety or depression symptoms were independently associated with postpartum levels of anxiety and depression symptoms [27]. They showed that life events related to health and sickness of self or loved ones, to the relation with the partner or conflicts with loved ones, or to work, finance, or housing problems were significantly associated with higher postpartum levels of anxiety and depression symptoms, adjusted for antenatal levels, suggesting that events during pregnancy not specifically related to the pregnancy and birth, are a highly important predictor for postpartum mental health. Kim et al. hypothesized that the association between symptoms of stress, depression, or anxiety during pregnancy and fetal development is stronger in pregnant women of high-risk compared to low-risk groups, defined by their socio-economic status. More specifically, they investigated the relationship between maternal depression, stress, positive and negative emotions, and maternal attachment to the fetus on estimated fetal body weight and biparietal diameter in late pregnancy, both in groups of women with high or low income [28]. Pregnant women in the low-income group showed higher levels of depression and stress compared to the high-income group at 16–20 weeks gestational age. Moreover, only the middle-income group showed a significant positive correlation between maternal-fetal attachment and negative emotion at 16–20 weeks gestational age that was associated with the biparietal diameter and fetal body weight at 33–35 weeks gestational age. A possible explanation is that women with a higher income have better access to high quality health care and are more likely to make healthier life choices. This emphasizes the need to monitor women at high risk more closely during their pregnancy to prevent and/or treat stress and depression and avert possible negative child outcomes. For psychiatric disorders, such as major depression disorder or generalized anxiety disorder, women can receive psychiatric and psychological evidence-based treatment, such as cognitive behavioral therapy (CBT). In an earlier pilot randomized controlled trial, CBT proved to be an effective method for reducing depression and anxiety symptoms in pregnant women. In a follow up of this trial, Bleker et al. investigated five years postpartum whether the children born from these pregnancies showed differences in brain characteristics compared to the children born to women who were allocated to the control group, and thus had not received CBT during pregnancy for depression and anxiety [29]. Results suggested a positive effect of prenatal CBT on cortical thickness, grey matter concentration, and white matter fiber cross-sections of fiber bundles that play a role in the stress response. However, it should be noted that the study was explorative and the findings require replication in larger studies. To effectively treat women who experience moderate to high levels of stress, easy-to-learn stress reducing techniques may be helpful. One such novel and promising method to reduce stress is heart rate variability (HRV)-biofeedback. The goal of HRV-biofeedback is to increase HRV through paced breathing exercises (about six breaths per minute). In order to do this, a small hand-held device measures the user’s heart rate, and the device uses this information to provide feedback on the optimal breathing frequency. Van der Zwan et al. investigated whether HRV-biofeedback is effective in reducing health complaints, including stress, anxiety, and depression, and in improving general well-being and sleep quality in both pregnant and non-pregnant women [30]. HRV-biofeedback had a significant positive effect on psychological well-being for all women and had an additional significant beneficial effect on anxiety complaints for pregnant women. Future research may focus on whether HRV-biofeedback aids in limiting adverse consequences of maternal anxiety during pregnancy on her offspring. In an opinion piece, we, Bleker, de Rooij and Roseboom, contemplate on the evidence for the consequences of low versus high prenatal stress for child neurodevelopment, and we discuss whether the message to pregnant women that stress is harmful for their child, should be attenuated. In our opinion, the many methodological challenges that are still hampering our knowledge about the actual effects of prenatal stress on child neurodevelopment should be acknowledged, and we believe that there are good reasons to not unnecessarily alarm women about potential negative effects of mild maternal stress during pregnancy on the developing fetal brain, based on (the gaps in) our current knowledge on this subject

## 4. Conclusions

Results from studies presented in this Special Issue on prenatal psychological stress and child neurodevelopment, emphasize the influence of socio-environmental and economic factors on perinatal mental health and hence the neurodevelopment of the (unborn) child. Preventive measures specifically targeting pregnant women at a high risk for developing perinatal depression, anxiety, and stress appear effective. In women who are already affected by depression, anxiety, or who suffer from chronic stress during their pregnancy, tailored interventions show promising results in clinical trials, with relatively low costs and a high usability. Moreover, the interventions for perinatal maternal mood may concurrently improve fetal and child development, potentially benefiting multiple generations. These interventions thus have the potential to provide answers to the question on how to deal with the global burden of adverse (perinatal) mental health. In a recent systematic review, quantitative and qualitative studies evaluating the clinical effectiveness, cost-effectiveness, safety, and acceptability of interventions to prevent postnatal depression were synthesized. The authors concluded that several interventions appear to be cost-effective relative to usual care, but are subject to considerable uncertainty, and that interventions warrant replication within randomized controlled trials (RCTs) [31]. As the results from the studies included in this Special Issue are promising, but overall based on small studies in a limited number of different settings, we also recommend replication of findings in larger clinical trials in different socio-economic settings to show whether these are indeed effective strategies to improve maternal mental health and neurodevelopment of the offspring.

## References

[1] Woody C.A., Ferrari A.J., Siskind D.J., Whiteford H.A., Harris M.G. (2017). A systematic review and meta-regression of the prevalence and incidence of perinatal depression. J. Affect Disord..

[2] Dennis C.L., Falah-Hassani K., Shiri R. (2017). Prevalence of antenatal and postnatal anxiety: Systematic review and meta-analysis. Br. J. Psychiatry.

[3] Loomans E.M., Van Dijk A.E., Vrijkotte T.G., Van Eijsden M., Stronks K., Gemke R.J., Van den Bergh B.R. (2013). Psychosocial stress during pregnancy is related to adverse birth outcomes: Results from a large multi-ethnic community-based birth cohort. Eur. J. Public Health.

[4] Phelan A.L., DiBenedetto M.R., Paul I.M., Zhu J., Kjerulff K.H. (2015). Psychosocial Stress during First Pregnancy Predicts Infant Health Outcomes in the First Postnatal Year. Matern. Child Health J..

[5] Yuksel F., Akin S., Durna Z. (2014). Prenatal distress in Turkish pregnant women and factors associated with maternal prenatal distress. J. Clin. Nurs..

[6] Hou Q., Li S., Jiang C., Huang Y., Huang L., Ye J., Pan Z., Teng T., Wang Q., Jiang Y. (2018). The associations between maternal lifestyles and antenatal stress and anxiety in Chinese pregnant women: A cross-sectional study. Sci. Rep..

[7] Tang X., Lu Z., Hu D., Zhong X. (2019). Influencing factors for prenatal Stress, anxiety and depression in early pregnancy among women in Chongqing, China. J. Affect Disord..

[8] Pantha S., Hayes B., Yadav B.K., Sharma P., Shrestha A., Gartoulla P. (2014). Prevalence of Stress among Pregnant Women Attending Antenatal Care in a Tertiary Maternity Hospital in Kathmandu. J. Women’s Health Care.

[9] Van den Bergh B.R., Mulder E.J., Mennes M., Glover V. (2005). Antenatal maternal anxiety and stress and the neurobehavioural development of the fetus and child: Links and possible mechanisms. A review. Neurosci. Biobehav. Rev..

[10] Glover V. (2014). Maternal depression, anxiety and stress during pregnancy and child outcome; what needs to be done. Best Pract. Res. Clin. Obstet. Gynaecol..

[11] Glover V. (2015). Prenatal stress and its effects on the fetus and the child: Possible underlying biological mechanisms. Adv. Neurobiol..

[12] Fisher J., Mello M.C.D., Patel V., Rahman A., Tran T., Holton S., Holmes W. (2012). Prevalence and determinants of common perinatal mental disorders in women in low- and lower-middle-income countries: A systematic review. Bull. World Health Organ..

[13] Gelaye B., Rondon M.B., Araya R., Williams M.A. (2016). Epidemiology of maternal depression, risk factors, and child outcomes in low-income and middle-income countries. Lancet Psychiatry.

[14] Chung E.K., Siegel B.S., Garg A., Conroy K., Gross R.S., Long D.A., Lewis G., Osman C.J., Messito M.J., Wade R. (2016). Screening for Social Determinants of Health among Children and Families Living in Poverty: A Guide for Clinicians. Curr. Probl. Pediatr. Adolesc. Health Care.

[15] Bogic M., Njoku A., Priebe S. (2015). Long-term mental health of war-refugees: A systematic literature review. BMC Int. Health Hum. Rights.

[16] McLean M.A., Cobham V.E., Simcock G., Kildea S., King S. (2019). Toddler Temperament Mediates the Effect of Prenatal Maternal Stress on Childhood Anxiety Symptomatology: The QF2011 Queensland Flood Study. Int. J. Environ. Res. Public Health.

[17] Faleschini S., Rifas-Shiman S.L., Tiemeier H., Oken E., Hivert M.F. (2019). Associations of Prenatal and Postnatal Maternal Depressive Symptoms with Offspring Cognition and Behavior in Mid-Childhood: A Prospective Cohort Study. Int. J. Environ. Res. Public Health.

[18] Lewis B.A., Gjerdingen D., Schuver K., Avery M., Marcus B.H. (2018). The effect of sleep pattern changes on postpartum depressive symptoms. BMC Women’s Health.

[19] Palagini L., Gemignani A., Banti S., Manconi M., Mauri M., Riemann D. (2014). Chronic sleep loss during pregnancy as a determinant of stress: Impact on pregnancy outcome. Sleep Med..

[20] Kamysheva E., Skouteris H., Wertheim E.H., Paxton S.J., Milgrom J. (2010). A prospective investigation of the relationships among sleep quality, physical symptoms, and depressive symptoms during pregnancy. J. Affect Disord..

[21] Bais B., Lindeboom R., van Ravesteyn L., Tulen J., Hoogendijk W., Lambregtse-van den Berg M., Kamperman A. (2019). The Impact of Objective and Subjective Sleep Parameters on Depressive Symptoms during Pregnancy in Women with a Mental Disorder: An. Explorative Study. Int. J. Environ. Res. Public Health.

[22] Emmanuel E., Creedy D.K., St John W., Gamble J., Brown C. (2008). Maternal role development following childbirth among Australian women. J. Adv. Nurs..

[23] Plews C., Bryar R., Closs J. (2005). Clients’ perceptions of support received from health visitors during home visits. J. Clin. Nurs..

[24] Leahy Warren P. (2005). First-time mothers: Social support and confidence in infant care. J. Adv. Nurs..

[25] Tarkka M.T. (2003). Predictors of maternal competence by first-time mothers when the child is 8 months old. J. Adv. Nurs..

[26] Milgrom J., Hirshler Y., Reece J., Holt C., Gemmill A.W. (2019). Social Support—A Protective Factor for Depressed Perinatal Women?. Int. J. Environ. Res. Public Health.

[27] Aris-Meijer J., Bockting C., Stolk R., Verbeek T., Beijers C., van Pampus M., Burger H. (2019). What If Pregnancy Is Not. Seventh Heaven? The Influence of Specific Life Events during Pregnancy and Delivery on the Transition of Antenatal into Postpartum Anxiety and Depression. Int. J. Environ. Res. Public Health.

[28] Kim D., Lee I., Bang K.S., Kim S., Yi Y. (2019). Do the Emotions of Middle-Income Mothers Affect. Fetal Development More Than Those of High-Income Mothers?—The Association between Maternal Emotion and Fetal Development. Int. J. Environ. Res. Public Health.

[29] Bleker L.S., Milgrom J., Parker D., Gemmill A.W., Holt C.J., Connelly A., Burger H., Roseboom T.J., de Rooij S.R. (2019). Brain Magnetic Resonance Imaging Findings in Children after Antenatal Maternal Depression Treatment, a Longitudinal Study Built on a Pilot Randomized Controlled Trial. Int. J. Environ. Res. Public Health.

[30] van der Zwan J.E., Huizink A.C., Lehrer P.M., Koot H.M., de Vente W. (2019). The Effect of Heart Rate Variability Biofeedback Training on Mental Health of Pregnant and Non-Pregnant Women: A Randomized Controlled Trial. Int. J. Environ. Res. Public Health.

[31] Morrell C.J., Sutcliffe P., Booth A., Stevens J., Scope A., Stevenson M., Harvey R., Bessey A., Cantrell A., Dennis C.L. (2016). A systematic review, evidence synthesis and meta-analysis of quantitative and qualitative studies evaluating the clinical effectiveness, the cost-effectiveness, safety and acceptability of interventions to prevent postnatal depression. Health Technol. Assess..

